# Postprandial energy metabolism and substrate oxidation in response to the inclusion of a sugar- or non-nutritive sweetened beverage with meals differing in protein content

**DOI:** 10.1186/s40795-017-0170-2

**Published:** 2017-07-21

**Authors:** Shanon L. Casperson, Clint Hall, James N. Roemmich

**Affiliations:** USDA, Agricultural Research Service, Grand Forks Human Nutrition Research Center, 2420 2nd Ave. North, Grand Forks, ND 58203-9034 USA

**Keywords:** Protein, Sugar-sweetened, Fat utilization, Substrate oxidation, Energy metabolism, Diet induced thermogenesis, Non-nutritive sweetened

## Abstract

**Background:**

The macronutrient composition of the diet may play a more important role in maintaining a healthy body weight and preventing obesity than previously thought. The primary goal of this research was to determine the extent to which the simple addition of a small serving of a sugar-sweetened beverage (SSB) to meals with different macronutrient compositions impacts appetite, energy metabolism and substrate oxidation.

**Methods:**

Appetite, energy metabolism and substrate oxidation were measured in 27 healthy weight adults (age = 23 ± 5 y; BMI = 23 ± 2 kg/m^2^) on two occasions in a room calorimeter after consuming a SSB or a non-nutritive-sweetened beverage (NNSB) with a standard (15%E) or high- (30%E) protein meal. Meal carbohydrate (CHO) content was adjusted to maintain equivalent calories for both study visits. All meals were composed of the same foods and provided 17 g of fat and 500 non-beverage calories. Study visits were separated by at least 1 week and menstruating females were studied during the luteal phase (Days 15–20). The effects of sex, protein level and beverage type and their interactions on satiety, appetite for foods with specific taste profiles, diet-induced thermogenesis (DIT) and rates of substrate oxidation were assessed using a 3-way Repeated Measures Analysis of Variance.

**Results:**

Increasing dietary protein decreased hunger and increased satiety. Males were hungrier and less satisfied with the meals than females. Increasing dietary protein also decreased the desire to eat something savory, salty and fatty and the males had a greater appetite for food with these taste profiles. Interestingly, there was no effect of sex, dietary protein or beverage type on the desire to eat something sweet. The inclusion of a SSB markedly suppressed DIT (2.42% ± 5.91%) and fat oxidation (9.87 ± 11.09 g).

**Conclusion:**

Appetite sensations, food preferences, energy expenditure and substrate oxidation are significantly altered in response to changes in meal macronutrient composition produced by modifications in the protein content of a meal and consumption of a SSB. Most notably, consumption of a SSB during a meal markedly reduces energy efficiency and fat oxidation independent of macronutrient composition.

**Trial registrations:**

ClinicalTrials.gov: NCT02211599, registered August 05, 2014.

**Electronic supplementary material:**

The online version of this article (doi:10.1186/s40795-017-0170-2) contains supplementary material, which is available to authorized users.

## Background

Obesity is an increasing problem, both in the United States and globally. Evidence suggests that changes in the macronutrient composition of a diet may play a more dynamic role in sustaining energy balance than simply counting calories. Concomitant with the increased prevalence of obesity, there has been a shift in the macronutrient composition of the American diet. In the National Health and Nutrition Examination Survey 1 (NHANES I, 1971–1974), the percentage of energy intake (%E) from carbohydrates was reported to be 44%E, protein 17%E and fat 37%E [1]. By NHANES 2013–2014, carbohydrates had increased to 49%E while protein and fat decreased to 16%E and 33%E, respectively [2]. This change in the macronutrient composition of the American diet has increased total energy intake by approximately 984 kJ per day [1, 2].

Indeed, the substitution of one macronutrient, particularly protein, for another can markedly affect both sides of the energy balance equation [1, 3]. On the expenditure side, studies of human bioenergetics have consistently reported that increasing dietary protein while maintaining energy intake produces a greater and more prolonged thermic effect and greater total energy expenditure [4]. Furthermore, dietary protein intake potentially increases fat oxidation by up to 50% [5]. On the intake side, protein intake may be regulated in that decreasing protein consumption stimulates an increase in energy intake in an attempt to maintain a constant absolute intake of dietary protein [1, 6, 7]. A 1.5%E decrease in dietary protein intake increases energy intake from carbohydrates and fats by 14%, perhaps in an attempt to increase protein intake from less protein-rich food sources [7]. In a 4-day in-patient ad libitum crossover feeding trial, a 5%E decrease in dietary protein intake produced a 12% increase in total energy intake [8]. The authors calculated that this was equivalent to a 4.5 kJ increase in non-protein foods for every 1 kJ decrease in habitual protein intake. Alternatively, a 1%E increase in dietary protein intake corresponded to ca 130 – 226 kJ decrease in daily energy intake dependent upon weight status and macronutrient substitution [1]. Therefore, the shift in the American diet towards greater carbohydrate intake and reduced dietary protein, may explain the increase in total energy intake over the last 50 years [1].

The observed increase in dietary carbohydrates has come primarily from added sugars, accounting for approximately 16%E of total energy intake [9]. The largest single source of added sugar and discretionary energy intake in the American diet is sugar-sweetened beverages (SSBs) [10]. In addition to increasing energy intake, SSBs may significantly affect postprandial fat oxidation. Stookey et al. recently reported that the addition of orange juice with a standard breakfast meal decreased fat oxidation compared to the same meal coupled with water [11]. This acute effect on net fat oxidation leads to preferential increases in visceral adipose tissue when consumed daily over long periods [12, 13]. Taken together, these results suggest that the consumption of SSBs may be contributing to weight gain by adding energy to the diet and reducing fat oxidation. However, it is not known whether the inclusion of a SSB with a high-protein meal offsets the beneficial effects of increased dietary protein on appetite, energy metabolism and fat oxidation. We hypothesize that compared to non-nutritive-sweetened beverage (NNSB) consumption, consuming a SSB with a meal will increase appetite and diet-induced thermogenesis (DIT) independent of dietary protein. We also hypothesize that consumption of a SSB will reduce postprandial fat oxidation and that this effect will be greater when consumed with a usual (15%E) protein meal compared to a higher (30%E) protein meal.

## Methods

### Participants

A total of 34 healthy weight (BMI 18 - 25 kg/m^2^) adults were recruited for participation. Of these, 5 participants withdrew prior to any study related procedures and 2 participants withdrew after completing the first study visit. The study was reviewed and approved by the University of North Dakota Institutional Review Board. Informed written consent was obtained for each participant prior the initiation of any study procedures. Exclusion criteria included: body mass index (BMI) > 25 kg/m2; percent body fat greater than or equal to 25% for males and 35% for females [14]; allergies to any of the study foods, more than a 10% change in body weight within the past 2 months; current or planned pregnancy; lactation; metabolic illness/disease; active cancer or in short-term remission (less than 3 years); infectious diseases; alcohol or drug abuse; tobacco use; presence of acute illness; taking medications known to affect energy expenditure and appetite. This trial is registered at https://clinicaltrials.gov as NCT02211599 on August 5, 2014.

#### Experimental protocol

All procedures were performed at the USDA Grand Forks Human Nutrition Research Center (GFHNRC) Metabolic Research Unit (MRU). The hypothesis was tested using a double-blind, randomized, cross-over design with beverage type (sugar vs non-nutritive sweetener) and protein level (15%E vs 30%E) treated as within-subject factors. Before starting, each participant completed a screening exam. This exam included height, weight, body composition (Bod-Pod; Cosmed, Chicago, IL), fasting glucose levels (Accu-Check Avivia, Indianapolis, IN) after a ≥ 12 h overnight fast, and a health history questionnaire. In addition, participants received detailed instruction on keeping a 3-day food diary to document eating patterns prior to each study visit. Participants were instructed to maintain their habitual eating habits and actives of daily living.

Participants received 15%E protein on one visit and 30%E protein on another visit. Participants were randomized as to which dietary protein level (15%E or 30%E) they received first. The same %E of carbohydrate, fat, and protein was consumed at both meals (breakfast, lunch) within a testing day. At each study visit, participants received the NNSB at one meal and the SSB at the other meal. The order of beverage type for each visit was counterbalanced across participants. So, for study visit 1, if the participant received the NNSB with the breakfast meal, they received the SSB with their lunch. The beverage order was then reversed for the participant’s second study visit. Each study visit was separated by 1 to 8 weeks, dependent upon the subject’s schedule and chamber availability. Females were measured during the luteal phase of their menstrual cycle to control for possible confounding effects of the menstrual cycle on energy expenditure [15]. Participants were instructed not to exercise for 48 to 72 h prior to their metabolic studies. Participants completed a 7-day physical activity assessment at each study visit. Participants were asked to circle activities that they performed during the last 7 days continually for at least 10 min. Activities included those perceived as both moderate and vigorous activities; such as jogging/running, swimming laps, bowling, basketball, dancing and stair climbing. Participants were then asked many how days during the past 7 days did they do a moderate or vigorous activity and how much time, on average, was spent doing the activities. The last question asked the participant to compare their activity over the past 7 days to their usual physical activity over the previous 3 months.

**Fig. 1:**
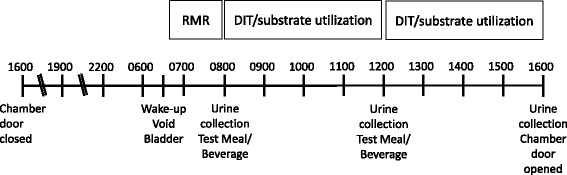
Experimental protocol

Figure 1 depicts the experimental protocol. Participants reported to the MRU at 1700 h the evening prior to the testing day. Each participant was weighed and orientated before entering the metabolic chamber. Exercise was not permitted while in the chamber. A non-study specific meal was provided at 1900 h. Water was provided ad libitum and consumption was measured throughout the 24 h chamber stay. At 2200 h the participant was instructed to turn off all electronic devices and prepare for bed. Lights out occurred at 2230 h. The following morning at 0700 h the participant was awakened and asked to void their bladder. Urine at this void was not collected. Participants were instructed to return to bed and assume a semi-recumbent position for the measurement of their resting metabolic rate (RMR; kcal/day). RMR was then measured for 30 to 45 min. Criteria for a valid RMR was a minimum of 20 min of steady state, determined as a < 10% fluctuation in oxygen consumption and <5% fluctuation in respiratory quotient. Urine was collected at 1200 h and 1600 h, as well as any voids occurring within those periods. Breakfast was provided at 0800 h and lunch was served at 1200 h. Immediately before and every 30 min after each meal participants rated their hunger, fullness, satiety, prospective food consumption, and desire to eat something sweet, salty, savory or fatty using a computer-based visual analogue scale (Sussex Ingestion Pattern Monitor, University of Sussex).

#### Meal compositions

Test meals were prepared and weighed by the GFHNRC research kitchen. Meal macronutrient composition is presented in Table 1. Diet 1 was formulated based on the U.S. Dietary Guidelines providing 55% carbohydrates, 30% fat and 15% protein. Diet 2 was protein-rich providing 40% carbohydrates, 30% fat, and 30% protein. All meals were comprised of the same foods and provided 500 non-beverage kcals. To minimize the monotony of consuming the exact same foods repeatedly, meals were presented differently for breakfast and lunch (Table 2). A 360 ml test beverage was served with each meal. The test beverage was made with water, black cherry powdered drink mix, and either sugar (31 g; 120 kcal) or a non-nutritive sweetener (sucralose; 4 g). Sucralose was matched to the sugar based on manufacturer conversions. Presenting the beverages in this way ensured that both beverages had the same flavor profile and level of sweetness. All test meals and beverages were consumed within 20 min.

**Table 1:** Macronutrient composition of the test meals

	Diet 1	Diet 2
Carbohydrate (g)	82.854	59.277
Protein (g)	22.806	44.624
Fat (g)	20.105	19.716

**Table 2:** Foods included in each test meal

Breakfast	%E Protein		Lunch	%E Protein	
	15% (g)	30% (g)		15% (g)	30% (g)
Ham Bake:			Ham sandwich:		
Diced potatoes	275	135	Bread – White	70	70
Diced ham	35	185	Sliced ham	35	185
Shredded cheddar cheese	20	20	Cheddar cheese slice	20	20
Bread - white	70	70	Potato wedges	275	135
Butter	12	5	Butter	12	5

#### 3-day food diaries

Participants completed a 3-day food diary prior to each study visit to estimate usual macronutrient intake. A Research Dietitian interviewed each participant during their study visits to determine completeness and accuracy of the food diary. Dietary intake was analyzed using the USDA National Nutrient Database for Standard Reference [16] and a customized in-house nutrient analysis program. The customized in-house nutrient analysis program (Grand Forks Research Analysis of Nutrient Data) is an interactive coding element that utilizes the USDA National Nutrient Database for Standard Reference for nutrient data [16]. The analysis program is not available for commercial use.

#### Specimen collection and analysis

Urine samples were pooled by time periods (morning and afternoon) and volumes were recorded. Specific gravity, protein content, and refractivity index were measured (Reichert, Inc. Depew, NY). Urine was aliquoted, frozen and stored for later nitrogen analysis after each collection period. Total nitrogen was determined using Dumas combustion method (rapid N Exceed; Elementar Americas Inc., Mt. Laurel, NJ).

#### Metabolic chamber

The metabolic chamber, designed by MEI Inc. (Minneapolis, MN), is a 3.6 m long, 3.0 m wide, and 2.2 m high room having a total volume of 25m^3^. The chamber is furnished with a bed, chair, desk, computer, cable television, wireless access, sink, and toilet. Walls and ceiling are constructed of 4 inch polyurethane foam panels sandwiched between steel sheets. The chamber has two windows providing outside views. A gasket-sealed aluminum frame door containing two transparent acrylic panels provides access to the chamber. The door and the two outside windows contribute to a sense of light and openness reducing anxiety. An airlock system allows meal trays and other materials to be passed to and from the participant. A ceiling mounted HVAC unit containing blowers for air circulation and particle filters maintains constant temperature and humidity in the chamber. The chamber is equipped with temperature, humidity, and barometric pressure sensors to monitor and provide continuous feedback for maintaining a stable environment. A dimmer switch allowed participants to control the brightness of the florescent ceiling lights. Microwave motion sensor (BB-150, Museum Technology Source Inc. Wilmington, MA) was used to detect participant movement. Two video cameras (2600 IP Camera, Cisco Systems Inc.; San Jose, CA), linked to monitors located in the nurses station of the MRU, provided remote monitoring of participant activity. A smoke detector in the chamber connected to the fire security system provided additional safety. Alarms were set to monitor oxygen (O_2_), carbon dioxide (CO_2_), temperature, and pressure in the chamber. Two curtains could be drawn across the door and toilet areas for privacy. When not needed the curtains were kept against the walls to prevent air flow restriction.

The metabolic chamber operates in a push-pull configuration. Both inflow and outflow O_2_ and CO_2_ concentrations are measured simultaneously allowing operation at lower ventilation rates, improving resolution, and response time. This configuration facilitates using doors and blood ports by producing minimal pressure differences between the chamber and outside air. Inflow and outflow rates were maintained by PID control. Initially both flow rates were kept low to bring O_2_ and CO_2_ to optimal levels for the gas analyzers and then were adjusted up or down as needed to maintain safe CO_2_ levels. Inflow and outflow rates were measured using thermal mass flow meters (HFM-D-301, Teledyne Hastings Instruments; Hampton, VA). Inflow rates were typically between 50 and 70LPM. A fraction of both inflow and outflow air was continuously withdrawn by pumps, filtered, and passed through a drying column (Perma Pure LLC; Toms River, NJ) before being analyzed. Inflow and outflow O_2_ and CO_2_ concentrations were measured using Ultramax/Oxymat 6 gas analyzers (Siemens AG; Nuremberg, Germany). The oxygen analyzers reference cells were supplied with a constant flowing gas having a concentration of 21% O_2_ and balance as nitrogen (N^2^).

Signals from analog sensors were routed into a DAQ (Measurement Computing, Norton MA), digitized and sent via USB bus to the controller PC. Flow meters and gas analyzers signals were sent to a National Instrument (Austin, TX) RS232 to USB digital converter then sent to the controller PC by USB bus. Data were logged at 60 s intervals. A backward derivative was used to reduce noise and smooth real-time graph data. Control and real-time monitoring of the chamber was accomplished using a custom template developed with National Instruments LabVIEW software. A 20 min “null” was recorded into the data file at the end of the chamber stay by switching valves so both in and out flows measured the same air source. The null measurement was subsequently used to correct for any minor drift between inflow and outflow analyzers that may have developed.

Custom certified gas mixtures (21% O_2_, balance N_2_; and 20% O_2_, 1% CO_2_, balance N_2_) were used to calibrate O_2_ and CO_2_ analyzers using a two point system. Calibration was checked prior to each participant visit and immediately afterwards to confirm that no analyzer drift occurred during testing. Validation was conducted monthly to ascertain functional status of the chamber. A custom built gas blender (MEI Inc., Minneapolis, MN) was used to infuse various blends of CO_2_ and nitrogen (N_2_) mimicking different metabolic conditions in the chamber for further validation.

#### Calculations and statistical analysis

Chamber data were imported into custom software (PiLR; MEI Research, Ltd.) for the analysis of energy metabolism and substrate oxidation. Briefly, average minute values of V̇_O2_ and V̇_CO2_ were recalculated using an 8 min center derivative and a Haldane filter. Periods of interest, such as resting and post meals, were set and average V̇_O2_, V̇_CO2_, EE, and RQ were determined. A null offset was calculated and applied to correct for differences between analyzers. Protein oxidation derived from urinary nitrogen was used to correct carbohydrate and fat oxidation and were calculated as follows:

$$ \mathrm{Protein}\ \mathrm{Oxidation}=\left({{\mathrm{N}}_2}^{\ast }6.26\right)/0.966 $$

$$ \mathrm{Carbohydrate}\ \mathrm{Oxidation}=\left({4.113}^{\ast }{{\dot{\mathrm{V}}}_{\mathrm{CO}2}}^{\mathrm{recalculated}}\right)\hbox{--} \left({2.907}^{\ast }{{\dot{\mathrm{V}}}_{\mathrm{O}2}}^{\mathrm{recalculated}}\right)\hbox{--} \left({3.75}^{\ast}\mathrm{Protein}\ \mathrm{Oxidation}\right) $$

$$ \mathrm{Fat}\ \mathrm{Oxidation}=\left({1.689}^{\ast }{{\dot{\mathrm{V}}}_{\mathrm{CO}2}}^{\mathrm{recalculated}}\right)\hbox{--} \left({1.689}^{\ast }{{\dot{\mathrm{V}}}_{\mathrm{O}2}}^{\mathrm{recalculated}}\right)\hbox{--} \left({0.324}^{\ast}\mathrm{Protein}\ \mathrm{Oxidation}\right) $$

For the calculation of DIT, energy expenditure and activity, as measured by Doppler radar, 30 min time periods after each meal were averaged and plotted to determine the y-intercept for each individual’s linear regression. DIT was then calculated as the difference between individual resting metabolic rates and their y-intercept [17]. DIT is also expressed as a percentage of the energy content of the meal for the 240 min postprandial period [18].

The effects of sex, protein level and beverage type and their interaction on satiety, appetite for specific taste profiles, DIT and rates of substrate oxidation were assessed using a 3-way Repeated Measures ANOVA. Multiple linear regression models were used to test whether habitual macronutrient and energy intake predicted metabolic responses to the test meals. Indicator variables were included in each model to test whether the relationship between intake and response differed by protein level and beverage type. Ratings of satiety and appetite for specific taste profiles, protein level, and beverage type were summarized by plotting the responses over time and calculating the area under the curve (AUC) using the trapezoid rule. Significance was set at *p* ≤ 0.05. The primary dependent variable, upon which the study is powered, is lipid oxidation. Power analyses demonstrated that 15 subjects provided greater than 90% power to detect a protein effect of 2 g on lipid oxidation [5] and a beverage effect of 5 g on lipid oxidation [11] given a within-subject SD of 2 g at *p* = 0.05. Secondary dependent variables included energy expenditure, DIT and protein and carbohydrate oxidation. Independent variables are sex (male, female), beverage type (SSB, NNSB) and protein level (15%E, 30%E). All statistical analysis was performed using SAS V9.4, SAS Institute, Inc., Cary, NC). Data are presented as meanSD unless otherwise noted.

## Results

### Participants

Twenty-seven healthy participants (13 male, 14 female) completed the study. Subject characteristics were age: 23 ± 5 yr., height: 173 ± 11 cm, weight: 69 ±12 kg, BMI: 23 ± 2 kg/m^2^, body fat: 20 ±6%, lean body mass (LBM): 54 ± 15 kg, fat mass (FM): 16 ±9 kg. There was no difference in habitual dietary intake before the two study visits. Daily energy consumption from the 3-day diet records for study visit 1 and 2 was 2215 ± 613 kcal/day and 2192 ± 514 kcal/day, respectively. The macronutrient composition of the participants’ usual diet was 44 ±10%E carbohydrates, 17 ±5%E protein and 36 ±7%E fat for study visit 1 and 45 ±7%E carbohydrates, 18 ±4%E protein and 37 ±5%E fat for study visit 2. Habitual protein consumption during the study period was 93 ± 27 g protein/day or approximately 1.36 ±0.34 g protein/kg/day. Subject characteristics by gender are provided in Additional file 1: Table S1.

Physical activity did not differ for the 7 days prior to each study visit. Prior to visit 1, participants reported 36 ±26 min on 2.4 ±1.8 days of moderate physical activity (MPA) and 17 ±19 min on 1.2 ±1.0 days of vigorous activity (VPA). Prior to their second study visit; participants reported doing 30 ± 23 min on 2.5 ±1.8 days of MPA and 25 ± 38 min on 1.2 ± 1.1 days of vigorous VPA.

### Substrate oxidation

**Fig. 2:**
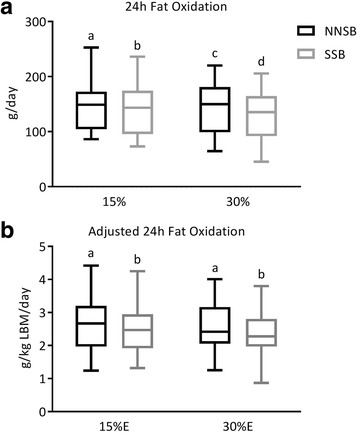
Postprandial fat oxidation. Absolute (**a**) and adjusted (**b**) fat oxidation in response to meals containing 15%E or 30%E protein with a sugar-sweetened beverage (SSB) or non-nutritive-sweetened beverage (NNSB) are presented as box and whickers plots with the line representing the median, the box representing the 25th to 75th percentiles and the whiskers representing the minimum to maximum values. There were significant main effects of sex and beverage type on absolute fat oxidation. Absolute fat oxidation was greater in the males compared to the females. There was no main effect of sex after adjustment for lean body mass (LBM). Fat oxidation was significantly reduced after consuming a SSB. There was no significant main effect of protein level nor were there any sex, protein level or beverage type interactions. Items with similar letters are not significant different

There was a main effect of sex (*p = *0.0043) and beverage type (*p = *0.0356) on postprandial fat oxidation. Postprandial fat oxidation was greater in the males (161 ± 44 g/day) compared to the females (119 ± 37 g/day). Consuming a SSB with a meal suppressed fat oxidation compared to NNSB consumption (135 ± 45 g/day and 145 ± 46 g/day, respectively). On average, postprandial fat oxidation decreased by 7.2 ±11 g and 12.6 ± 11 g with the addition of a SSB to a meal (15% and 30% protein, respectively). There was no significant main effect of protein amount nor were there any significant interactions between sex, protein amount, or beverage type on postprandial fat oxidation (Fig. 2A **and** Table 3).

**Table 3:** Postprandial energy expenditure and macronutrient oxidation

	Males				Females			
	15%E-NNSB	15%E-SSB	30%E-NNSB	30%E-SSB	15%E-NNSB	15%E-SSB	30%E-NNSB	30%E-SSB
EE (kcal/day)	2641 ± 263^a^	2772 ± 283^b^	2714 ± 295^a^	2724 ± 291^b^	2109 ± 192^c^	2181 ± 270^d^	2070 ± 197^c^	2173 ± 223^d^
EE (kcal/kg LBM/day)	40 ± 5^a^	42 ± 5^b^	41 ± 4^a^	41 ± 4^b^	44 ± 3^c^	46 ± 6^d^	44 ± 4^c^	46 ± 5^d^
DIT (kcal/min)	0.36 ± 0.16^a^	0.40 ± 0.15^a^	0.41 ± 0.16^a^	0.42 ± 0.15^a^	0.33 ± 0.11^a^	0.35 ± 0.12^a^	0.39 ± 0.13^a^	0.40 ± 0.07^a^
DIT (% intake)	17 ± 8^a^	16 ± 6^b^	20 ± 8^a^	16 ± 6^b^	16 ± 5^a^	14 ± 5^b^	18 ± 6^a^	16 ± 3^b^
Carbohydrate oxidation (g/day)	237 ± 75^a^	278 ± 75^b^	247 ± 61^a^	291 ± 66^b^	203 ± 68^a^	246 ± 60^b^	215 ± 83^a^	253 ± 81^b^
Protein oxidation (g/day)	20 ± 5^a^	22 ± 9^a^	24 ± 8^b^	23 ± 10^b^	14 ± 4^c^	15 ± 3^c^	18 ± 5^d^	19 ± 6^d^
Protein oxidation (g/kg LBM/day)	0.31 ± 0.08^a^	0.33 ± 0.13^b^	0.36 ± 0.13^a^	0.35 ± 0.15^b^	0.30 ± 0.10^a^	0.30 ± 0.06^b^	0.38 ± 0.11^a^	0.38 ± 0.11^b^
Lipid oxidation (g/day)	166 ± 47^a^	161 ± 43^b^	168 ± 44^a^	151 ± 43^b^	130 ± 434^c^	119 ± 39^d^	119 ± 39^c^	111 ± 38^d^
Lipid oxidation (g/kg LBM/day)	2.53 ± 0.73^a^	2.45 ± 0.69^b^	2.52 ± 0.62^a^	2.26 ± 0.59^b^	2.72 ± 0.72^a^	2.50 ± 0.83^b^	2.58 ± 0.89^a^	2.40 ± 0.84^b^

Values are mean ± SD

*LBM* lean body mass

Items with similar letters are not significantly different

Postprandial fat oxidation was positively correlated (*r* = 0.48; *p* < 0.0001) with lean body mass (LBM; Additional file 2: Figure S1). When expressed relative to LBM (g/kg LBM/day), there was a main effect of beverage type (*p* = 0.0420). Adjusted fat oxidation was greater after consuming a NNSB (2.6 ± 0.7 g/kg LBM/day) with a meal compared to a SSB (2.4 ± 0.7 g/kg LBM/day). There were no significant main effects of sex or protein amount nor were there any significant interactions between sex, protein amount, or beverage type on adjusted postprandial fat oxidation (Fig. 2B **and** Table 3). There was no significant correlation between postprandial fat oxidation and FM (Additional file 2: Figure S2).

There was a main effect of sex (*p* = 0.0056) and protein level (*p* = 0.0013) on postprandial protein oxidation. Protein oxidation was greater in the males (22 ± 8 g/day) compared to the females (16 ± 5 g/day). Protein oxidation was greater with the 30%E (21 ± 8 g/day) compared to the 15%E (18 ± 6 g/day) protein meal. There was no significant main effect of beverage type nor were there any significant interactions between sex, protein amount, or beverage type on postprandial protein oxidation (Table 3).

Protein oxidation was positively correlated (*r* = 0.32; *p* = 0.0010) with LBM (Additional file 2: Figure S1). When expressed relative to LBM (g/kg LBM/day), there was a main effect of the amount of dietary protein contained in the meal (*p* = 0.0012). Adjusted postprandial protein oxidation was greater after consuming a NNSB (0.338 ± 0.112 g/kg LBM/day) with a meal compared to a SSB (0.340 ± 0.123 g/kg LBM/day). There were no significant main effects of sex or beverage type nor were there any significant interactions between sex, protein amount, or beverage type on adjusted postprandial protein oxidation. There was no significant correlation between postprandial protein oxidation and FM (Additional file 3: Figure 2).

There was a main effect of beverage type (*p* < 0.0001) on postprandial carbohydrate oxidation. Carbohydrate oxidation was greater after SSB (271 ± 76 g/day) compared to NNSB (231 ± 79 g/day) consumption with a meal. There were no significant main effects of sex or the amount of dietary protein in the meal nor were there any significant interactions between sex, protein amount, or beverage type on postprandial carbohydrate oxidation (Table 3).

Postprandial carbohydrate oxidation was positively correlated with LBM (*r* = 0.28; *p* = 0.0035; Additional file 2: Figure S1) and negatively correlated with FM (*r* = −0.32; *p* = 0.0010; Additional file 3: Figure S2). When expressed relative to LBM or FM, there was a main effect of beverage type (*p* = 0.0002). Adjusted carbohydrate oxidation was greater after consuming a SSB (4.7 ± 1.4 g/kg LBM/day and 22.9 ± 12.8 g/kg FM/day) with a meal compared to a NNSB (4.0 ± 1.4 g/kg LBM/day and 19.8 ± 12.3 g/kg FM/day). There were no significant main effects of sex or protein amount nor were there any significant interactions between sex, protein amount, or beverage type on adjusted postprandial carbohydrate oxidation.

### Energy metabolism

**Fig. 3:**
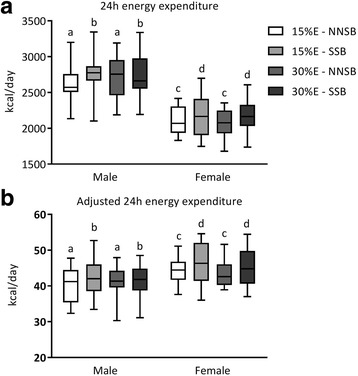
Twenty-four hour energy expenditure (EE). 24 h EE (**a**) and 24 h EE adjusted for lean body mass (LBM) (**b**) in response to meals containing 15%E or 30%E protein with a sugar-sweetened beverage (SSB) or non-nutritive-sweetened beverage (NNSB) are presented as box and whickers plots with the middle horizontal line representing the median, the box bottom and top representing the 25th to 75th percentiles and the whiskers representing the minimum to maximum values. There were significant main effects of sex and beverage type. Absolute 24 h EE (**a**) was greater in the males. After adjustment for LBM (**b**) 24 h EE was greater in the females. 24 h EE was greater after consuming a SSB. There was no significant main effect of protein level nor were there any sex, protein level or beverage type interactions. Items with similar letters are not significant different

There was a main effect of sex (*p* < 0.0001) and beverage type (*p* = 0.0004) on estimated 24 h energy expenditure (EE). As expected, estimated 24 h EE was greater in males (2713 ± 290 kcal/day) than females (2133 ± 230 kcal/day) and greater after SSB consumption (2463 ± 395 kcal/day) compared to NNSB consumption (2383 ± 384 kcal/day). There was no significant main effect of protein amount nor were there any significant interactions between sex, protein amount, or beverage type on 24 h EE (Fig. 3A **and** Table 3).

Estimated 24 h EE was positively correlated (*r* = 0.80; *p* < 0.0001) with LBM (Additional file 4: Figure S3). When expressed relative to LBM, there a main effect of sex (*p* = 0.0470) and beverage type (*p* = 0.0006). Adjusted 24 h EE was greater in females (45 ± 5 kcal/kg LBM/day) than males (41 ± 5 kcal/kg LBM/day) and greater after SSB consumption (44 ± 6 kcal/kg LBM/day) compared to NNSB consumption (42 ± 4 kcal/kg LBM/day). There was no significant main effect of protein amount nor were there any significant interactions between sex, protein amount, or beverage type on adjusted 24 h EE (Fig. 3B **and** Table 3).

There was a main effect of beverage type (*p* = 0.0240) on DIT. DIT was greater when participants consumed a NNSB (18% ± 7%) with a meal compared to a SSB (15% ± 5%). There were no significant main effects of sex or protein amount nor were there any significant interactions between sex, protein amount, or beverage type on DIT (Table 3). Although not significant, there was a trend (*p* = 0.0690) for the amount of dietary protein contained in the meal to increase DIT.

### Effect of habitual macronutrient intake on metabolic responses

Habitual carbohydrate intake did not predict carbohydrate oxidation (*F*(3102) = 0.24, *p* = 0.86, R^2^ = 0.007). There was a significant linear relationship between habitual fat intake and fat oxidation (*F*(3102) = 7.60, *p* < 0.0001, R^2^ = 0.18), protein intake and protein oxidation (*F*(3104) = 4.89, *p* = 0.003, R^2^ = 0.12) and energy intake and energy expenditure (*F*(3104) = 8.19, *p* < 0.0001, R^2^ = 0.19); however, the slopes did not vary by the protein level or beverage type in the test meals (fat: *p* = 0.81, protein: *p* = 0.78, energy: *p* = 0.76).

### Subjective appetite responses

**Fig. 4:**
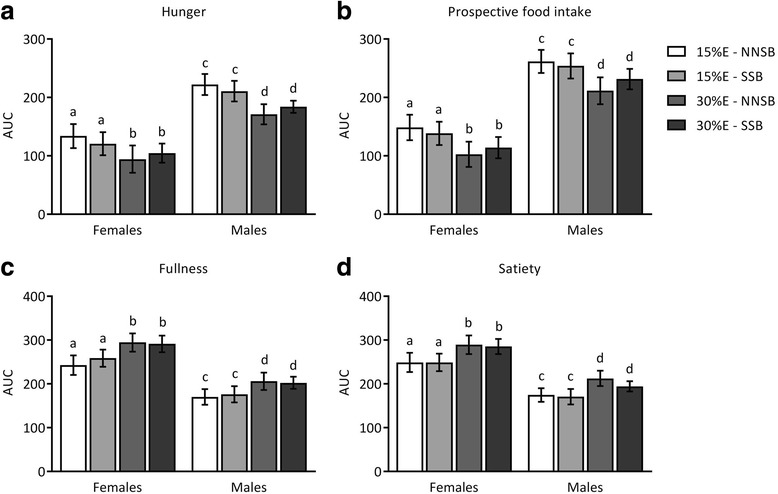
Appetite sensation scores. Subjective appetite sensations of hunger (**a**), prospective food intake (**b**), fullness (**c**), and satiety (**d**) after meals containing 15%E or 30%E protein with a sugar-sweetened beverage (SSB) or a non-nutritive-sweetened beverage (NNSB) are presented as area under the curve (AUC). Males reported feeling hungrier and that they could eat more food. Conversely, females reported greater feelings of fullness and satiety. Hunger and satiety AUC were lower after consuming a meal of 30%E protein. There was no significant main effect of beverage nor were there any sex, protein level or beverage type interactions. Items with similar letters are not significantly different. Data are presented as the mean ± SE

Figure 4 depicts the area under the curve (AUC) for subjective appetite sensations. There were significant main effects of sex and dietary protein for all subjective postprandial appetite sensations. Overall, the males reported greater hunger (*p* = 0.0007) and prospective food intake (*p* < 0.0001) and reduced fullness (*p* = 0.0015) and satiety (*p* = 0.0013). Increasing dietary protein intake from 15%E to 30%E decreased participant’s perception of their hunger (*p* < 0.0001) and prospective food intake (*p* < 0.0001), while increasing their perception of fullness (*p* < 0.001) and ratings of satiety (*p* < 0.0001). There was no significant main effect of beverage type nor were there any significant interactions between sex, protein amount, or beverage type on subjective postprandial appetite sensations.

**Fig. 5:**
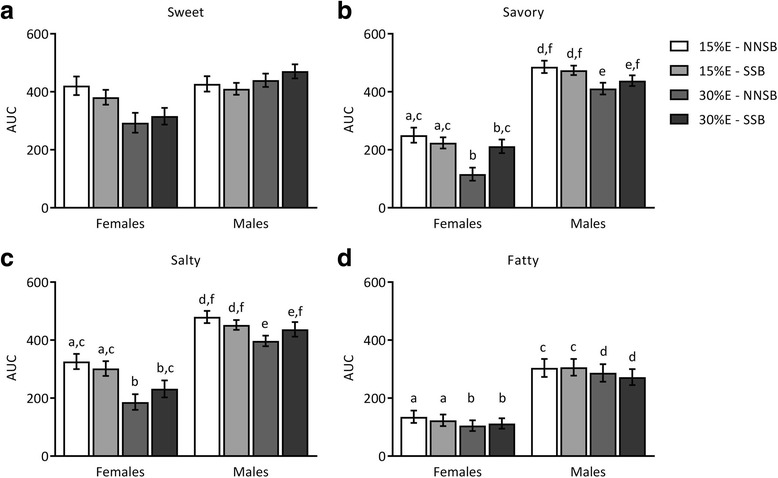
Appetite scores for foods with specific taste profiles. Subjective appetite scores for sweet (**a**), savory (**b**), salty (**c**), and fatty (**d**) tasting foods after meals containing 15%E or 30%E protein with a sugar-sweetened beverage (SSB) or a non-nutritive-sweetened beverage (NNSB) are presented as area under the curve (AUC). There were no significant main or interaction effects of sex, dietary protein or beverage type on the appetite for sweet food. There were significant main effects of sex and dietary protein and a protein level x beverage type interaction for the appetite for savory and salty foods. There were significant main effects of sex and dietary protein on the appetite for fatty foods. Items with similar letters are not significantly different. Data are presented as the mean ± SE

Figure 5 depicts the AUC for the appetite for foods with specific taste profiles. The desire to eat something sweet was not affected by sex, protein amount or beverage type. There was a significant sex x protein level interaction (*p* = 0.0113); however, post-hoc analysis did not reveal any pair-wise significance. On the other hand, there was a main effect of sex on the desire to eat something savory (*p* < 0.0001), salty (*p* = 0.0090) and fatty (*p* = 0.0091) with no significant interactions. The amount of dietary protein in the preceding meal influenced the desire to eat something savory (*p* = 0.0011), salty (*p* < 0.0001) and fatty (*p* = 0.0188). Increasing dietary protein markedly lowered the desire to consume these types of foods. In addition, there was a significant protein level x beverage type interaction for the desire to eat something savory and salty (*p* ≤ 0.05). Post-hoc analysis revealed that the primary effect of this interaction was between meals containing 15%E protein consumed with a NNSB compared to 30%E protein consumed with a NNSB.

## Discussion

The primary goal of this research was to determine the extent to which the addition of a SSB to standardized meals differing in dietary protein impacts appetite, energy metabolism and substrate oxidation. We found that SSB consumption modifies meal-induced alterations in food preferences, energy expenditure and substrate oxidation, thus, impacting both sides of the energy balance equation. On the intake side, the additional energy intake from the SSB did not influence satiety and the desire to eat savory and salty foods was increased when paired with a protein-rich meal. On the expenditure side, SSB consumption increased energy expenditure by 80 kcal, thus, creating a 40 kcal excess which was independent of dietary protein. SSB also decreased postprandial fat oxidation by 8%. These results highlight the impact SSB consumption can have on energy balance and substrate oxidation and provides further insight into the potential role of SSBs in the etiology of obesity.

The satiating effects of increasing dietary protein are well documented (see review [19]). As expected, increasing protein intake from 15%E to 30%E significantly reduced subjective ratings of hunger and prospective food intake, and increased ratings of fullness and satiety. The addition of a SSB to the meal did not further alter appetite sensations. These results are in line with both acute [20–22] and chronic [23, 24] studies showing little effect of sweetener type, especially when consumed in liquid form, on appetite sensations. In addition, dietary protein influenced food preferences in that increasing protein intake decreased the appetite for savory, salty and fatty foods. Consuming a SSB with a higher protein meal produced an interactive effect on food preferences in that it increased the appetite for savory and salty foods. Both animals and humans learn that specific taste profiles provide a general representation of the nutrient content of the food being consumed. Foods with a savory taste indicate a source of protein, a salty taste is associated with the protein and sodium content of a food, a sweet taste signifies a source of simple sugars [25], and recent evidence supports a possible taste component for dietary fats [26]. Interestingly, the present study found a decrease in the appetite for fatty foods with the protein-rich meal. If this truly reflects an alteration in the appetite for fat, this study demonstrates, for the first time, that increasing dietary protein could potentially decrease fat intake from other food sources. Additional studies are needed to elucidate a possible relationship between dietary protein and fat intake. The observed differences in the profiles for the appetite for savory and salty foods may be a compensatory response to decreasing the absolute amount of dietary protein and the dilution of the relative amount of dietary protein with the addition of a SSB [6, 7]. A limitation to this study is that we did not include an ad libitum meal at the end of the study period. However, these appetitive data support previous research showing a greater intake of savory (higher protein) foods in response to lower dietary protein intake [8, 27, 28]. Further research is needed to determine if simply including a SSB with a meal will also result in an increase in ad libitum protein intake.

The effect of SSB consumption on energy intake has received much attention [29–31]. Nevertheless, few studies have examined alterations in energy expenditure in response to SSBs. This is the first study to our knowledge to look at whether the inclusion of a single serving size of a SSB ingested with standardized meals differing in protein content affects energy metabolism. Inclusion of a SSB increased estimated 24 h EE, yet, DIT (as a percentage of energy intake) decreased. Interestingly, this negative effect of SSB consumption on DIT was greater with the protein-rich meal compared to the standard meal. The current quantitative thermogenesis data are in agreement with other reports showing an increase in EE after ingestion of sucrose compared to an equal serving of unsweetened pregelantinized corn starch [32] or a non-nutritive sweetener [33]. On the other hand, these results conflict with those reported by Prat-Larquemin et al. [33] showing no difference between sucrose and a non-nutritive sweetener (aspartame; 0.27 g) on DIT. The contradictory results could be attributed to differences in meal composition. The current study added a SSB or NNSB to standardized mixed meals compared to sweetening a single food item. In addition, the energy content of the solid foods was not adjusted in order to maintain isocaloric conditions between the sugar-sweetened and the non-nutritive sweetened trials. This allowed measurement of the effects of a SSB as it is typically consumed. The present results show that, although there is an increase in overall daily EE with SSB consumption, not all of the additional calories provided by the SSB are expended. This small shift in the energy balance equation, if no further adjustments are made in energy intake or expenditure through increased activity, may help explain the effect of SSB consumption on weight gain [34].

The reciprocal relationship between carbohydrate and fat oxidation is well known (see review [35]). In this study, carbohydrate oxidation increased in line with the additional carbohydrates supplied by the SSB, independent of meal macronutrient composition. On the other hand, there was almost a twofold difference in the change in fat oxidation when the SSB was consumed with the standard protein meal (7 g) compared to the protein-rich meal (13 g). Bortolotti et al. recently reported a greater suppression of fat oxidation when fructose was combined with a higher protein meal compared to fructose supplementation alone [36]. Because fructose is almost completely metabolized in the liver, and fat oxidation and de novo lipogenesis share the same metabolic pathways in the liver, it has been posited that the suppression of fat oxidation is the result of increased de novo lipogenesis [37]. In both animal and human trials, the fructose component of sucrose, but not the glucose, increases fractional de novo lipogenesis [38]. Other studies have shown that the long-term effect of increased carbohydrate intake mediates lipogenesis rather than oxidation [35]. Therefore, the suppression of fat oxidation with repeated SSB consumption, especially when paired with high-protein meals, over time could potentially lead to a greater tendency to store fat and, thus, increase body weight [35, 39].

The primary strength of this study was the control of macronutrients and macronutrient subtypes. Consumption of the same foods at each meal provided the opportunity to determine the impact of SSB consumption on appetite, EE and macronutrient partitioning. In addition, the whole-room calorimeter allowed for the precise measurement of EE and substrate utilization in response to minimal dietary changes. This study is not without limitations. First, only healthy weight adults were recruited for participation. It is possible that overweight and obese individuals may respond differently to the changes in meal macronutrient composition. However, the current study of healthy weight adults provides a basic indication of how SSB consumption can alter energy metabolism. Second, the data are for single test meals with a high glycemic index. Nonetheless, the foods used in the study permit external validity as they are most often the foods consumed by the general public (potatoes and white bread). Caution must be used when extrapolating these data to dietary changes over a long period. Finally, we did not control for the macronutrient composition of the habitual diet. Our 3-day diet records show that participants consumed a typical American diet based on current NHANES data [2] and did not significantly vary from one visit to the next. Additionally, the metabolic responses to the test meals were the same when habitual macronutrient intakes were used as a covariate.

## Conclusions

The present results demonstrate the effect of SSB consumption on energy metabolism and food choices. These results reject our hypothesis that increasing dietary protein would correspond to a diminution in the effects of SSB consumption. This work adds to the mounting evidence that SSB consumption can increase an individual’s susceptibility to weight gain and fat accumulation, especially when paired with a higher protein meal. These data highlight the need to design strategies aimed at maximizing macronutrient balance instead of focusing on interventions that strictly target energy balance.

## Additional files

Additional file 1: Table S1. Subject characteristics and habitual dietary intake. Values are expressed as mean ± SD. (DOCX 18 kb)

Additional file 2: Figure S1. Correlation between 24 h energy expenditure and lean body mass (top) and fat mass (bottom). Each dot represents a study participant. 24 h energy expenditure was positively correlated with lean body mass. There was no significant correlation between 24 h energy expenditure and fat mass. (JPG 227 kb)

Additional file 3: Figure S2. Correlation between substrate oxidation and lean body mass. Each dot represents a study participant. Fat (top), protein (middle) and carbohydrate (bottom) oxidation were positively correlated with lean body mass. (JPG 220 kb)

Additional file 4: Figure S3. Correlation between substrate oxidation and fat mass. Each dot represents a study participant. Fat (top) and protein (middle) oxidation were not significantly correlated with fat mass. Carbohydrate oxidation (bottom) was negatively correlated with lean body mass. (JPG 156 kb)

## Abbreviations

SSB: Sugar-sweetened beverage
NNSB: Non-nutritive sweetened beverage
LBM: Lean body mass
FM: Fat mass
DIT: Diet induced thermogenesis
EE: Energy expenditure
AUC: Area under the curve
ANOVA: Analysis of variance
