# Supplementary material for: Postprandial energy metabolism and substrate oxidation in response to the inclusion of a sugar- or non-nutritive sweetened beverage with meals differing in protein content
Source: BMC Nutr. 2017 Jul 21;3:49. doi: 10.1186/s40795-017-0170-2 (PMC7050861; doi:10.1186/s40795-017-0170-2)
Supplement: Supplementary file 1 — Subject characteristics and habitual dietary intake. Values are expressed as mean ± SD. (DOCX 18 kb) [file 40795_2017_170_MOESM1_ESM.docx]

**Table S1**: Subject characteristics and habitual dietary intake

|  | **All** | **Women** | **Men** | **P** |
| --- | --- | --- | --- | --- |
| n | 29 | 16 | 13 |  |
| Age (y) | 23 ± 5 | 24 ± 7 | 22 ± 2 | 0.310 |
| Weight (kg) | 69 ± 12 | 61 ± 8 | 78 ± 11 | 0.002 |
| Height (m) | 173 ± 11 | 168 ± 9 | 180 ± 9 | < 0.0001 |
| BMI (kg/m²) | 23 ± 2 | 22 ± 2 | 24 ± 2 | 0.002 |
| Fat mass (%) | 20 ± 6 | 24 ± 5 | 15 ± 5 | < 0.0001 |
| Fat mass (kg) | 16 ± 9 | 22 ± 15 | 12 ± 5 | 0.030 |
| Lean body mass (kg) | 54 ± 15 | 40 ± 15 | 65 ± 9 | < 0.0001 |
| **Habitual Dietary Intake** |  |  |  |  |
| Energy intake (Kcal/day) | 2167 ± 517 | 2024 ± 567 | 2370 ± 808 | 0.055 |
| Carbohydrate (g/day) | 243 ± 67 | 248 ± 95 | 239 ± 92 | 0.784 |
| Carbohydrate (%E) | 45 ± 8 | 48 ± 8 | 41 ± 5 | 0.003 |
| Protein (g/day) | 93 ± 27 | 78 ± 24 | 112 ± 40 | 0.0001 |
| Protein (%E) | 17 ± 4 | 16 ± 3 | 19 ± 4 | 0.007 |
| Fat (g/day) | 88 ± 29 | 79 ± 27 | 101 ± 48 | 0.031 |
| Fat (%E) | 36 ± 5 | 35 ± 4 | 38 ± 6 | 0.165 |
| Data are means ± SD. Student’s t-test used to determine gender differences. | | | | |
