# Supplementary figures and images for: Postprandial energy metabolism and substrate oxidation in response to the inclusion of a sugar- or non-nutritive sweetened beverage with meals differing in protein content
Source: BMC Nutr. 2017 Jul 21;3:49. doi: 10.1186/s40795-017-0170-2 (PMC7050861; doi:10.1186/s40795-017-0170-2)

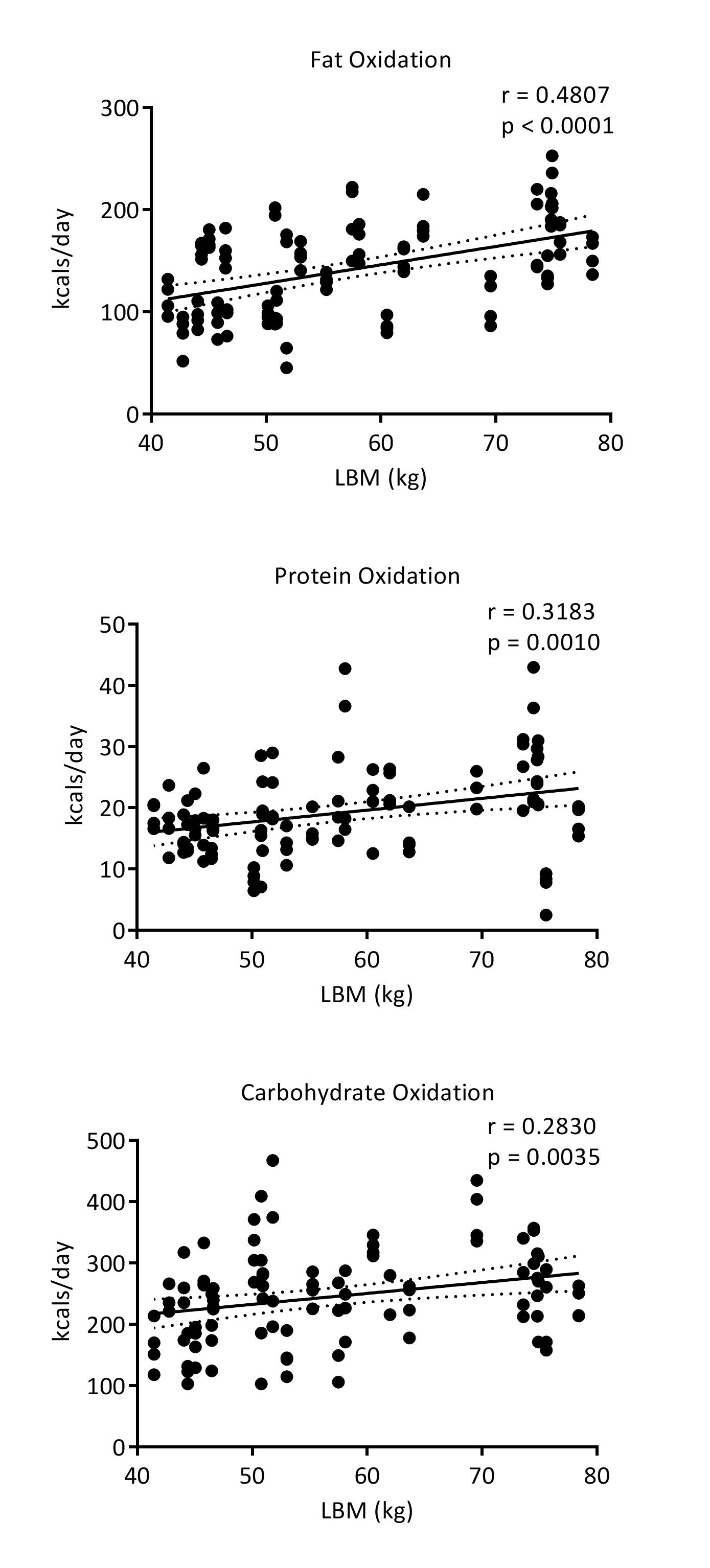

Supplement: Supplementary file 2 — Correlation between 24 h energy expenditure and lean body mass (top) and fat mass (bottom). Each dot represents a study participant. 24 h energy expenditure was positively correlated with lean body mass. There was no significant correlation between 24 h energy expenditure and fat mass. (JPG 227 kb) [file 40795_2017_170_MOESM2_ESM.jpg]

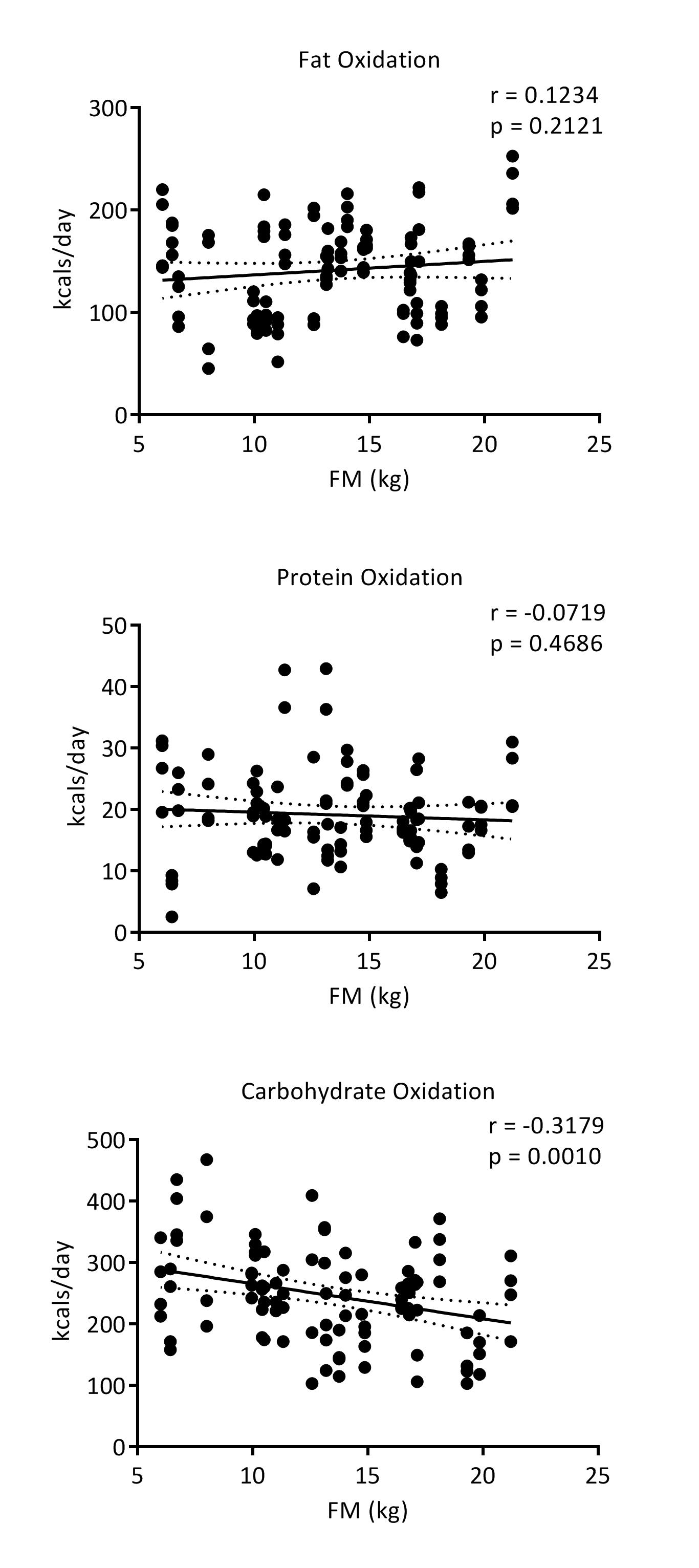

Supplement: Supplementary file 3 — Correlation between substrate oxidation and lean body mass. Each dot represents a study participant. Fat (top), protein (middle) and carbohydrate (bottom) oxidation were positively correlated with lean body mass. (JPG 220 kb) [file 40795_2017_170_MOESM3_ESM.jpg]

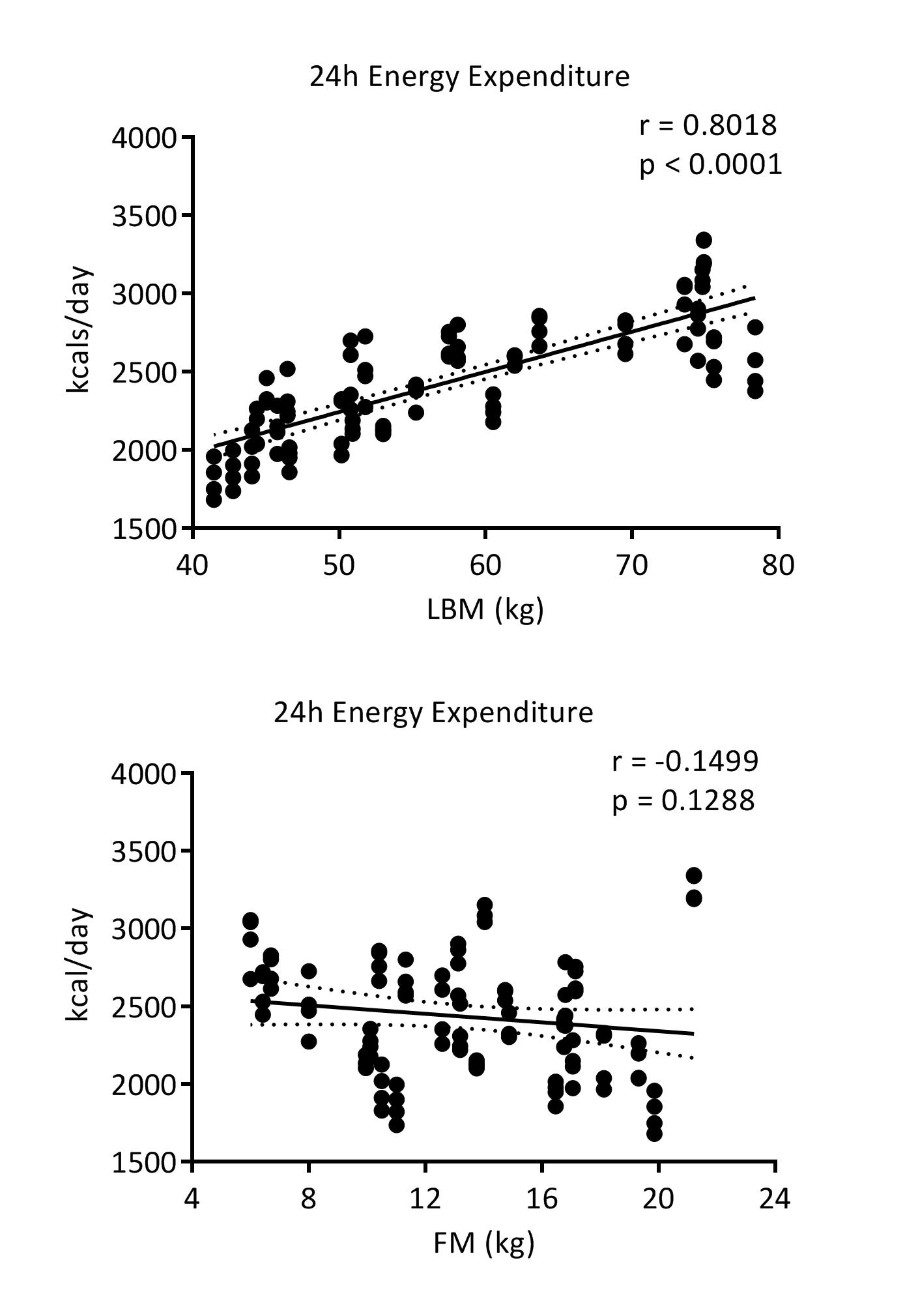

Supplement: Supplementary file 4 — Correlation between substrate oxidation and fat mass. Each dot represents a study participant. Fat (top) and protein (middle) oxidation were not significantly correlated with fat mass. Carbohydrate oxidation (bottom) was negatively correlated with lean body mass. (JPG 156 kb) [file 40795_2017_170_MOESM4_ESM.jpg]
